# Artificial intelligence-driven body surface area (BSA) estimation using computed tomography: Comparative evaluation with existing formulae

**DOI:** 10.1097/MD.0000000000048478

**Published:** 2026-04-24

**Authors:** Ronnie Sebro, Mahmoud Elmahdy

**Affiliations:** a Department of Epidemiology, MD Anderson Cancer Center, University of Texas, 1515 Holcombe Blvd, Unit 1475, Houston, TX; b Department of Imaging Physics, MD Anderson Cancer Center, University of Texas, 1515 Holcombe Blvd, Unit 1475, Houston, TX; c Department of Musculoskeletal Radiology, MD Anderson Cancer Center, University of Texas, 1515 Holcombe Blvd, Unit 1475, Houston, TX; d Department of Otolaryngology, University of South Florida, 12901 Bruce B. Downs Blvd, MDC 73, Tampa, FL.

**Keywords:** body surface area, dose, equations, pharmacology, race/ethnicity

## Abstract

Total body surface area (BSA) is thought to be a better predictor of metabolic mass than body weight because it is less affected by abnormal adipose mass, and as a result, it is often used for drug dosing in clinical medicine. There are several formulae used for estimating BSA, however, most are based on 2-dimensional and not 3-dimensional BSA measurements or used naïve statistical techniques to create the estimating formulae. This study analyzed 3-dimensional whole-body positron emission tomography/computed tomography studies from 698 patients. A limitation of this dataset was the imbalance in racial distribution. BSA was obtained from the computed tomography component of these studies using TotalSegmentator software. There was a positive correlation between BSA and height (*R* = 0.49, *P* < .001), and between BSA and weight (*R* = 0.83, *P* < .001). Multivariable ridge regression with 5-fold cross-validation was used to create new formulae to estimate BSA by adjusting for sex, race, height, and weight. The optimal formula to estimate BSA in m^2^, is $BSA=0.2626943*H^{0.280306}W^{0.415072}\left(1.001269\;if\;Male\right)\left(0.96941\;if\;White\right)$, where *H* is height in meters and *W* is weight in kilograms. Our formulae were significantly more accurate than all other formulae and had a lower mean absolute error and mean squared error than the Dubois (*P* < .001, *P* < .001), Mosteller (*P* < .001, *P* < .001), Haycock (*P* < .001, *P* < .001), Gehan (*P* < .001, *P* < .001), Boyd (*P* < .001, *P* < .001), Fujimoto (*P* < .001, *P* < .001), Takahira (*P* < .001, *P* < .001), Shuter and Aslani (*P* < .001, *P* < .001), Lipscombe (*P* < .001, *P* < .001), and Schlich (*P* < .001, *P* < .001) BSA formulae respectively.

## 1. Introduction

Total body surface area (BSA) is thought to be more highly correlated with metabolic mass than body weight and as a result, is often used for determining drug dosage in medicine.^[1]^ BSA is thought to be less affected by central and peripheral adiposity compared to body mass index (BMI).^[1,2]^ There are several formulae used for estimating BSA, including formulae by DuBois,^[3]^ Mosteller,^[4]^ Haycock,^[5]^ Gehan and George,^[6]^ Boyd,^[7]^ Fujiomoto,^[8]^ Takahira,^[8]^ Shuter and Aslani,^[9]^ Lipscomb,^[10]^ and Schlick.^[11]^ These formulae are between 5 and 120 years old.^[3–11]^ The formulae were also created crude 2-dimensional estimates of the true BSA.^[3–11]^ Therefore, there is a need to validate these formulas using modern technology, which allows us to have 3-dimensional measurements of the BSA.

Recent advances in medicine have allowed for high resolution total body imaging.^[12]^ Whole-body positron emission tomography/computed tomography (PET/CT) imaging is commonly used for staging and surveillance of patients with malignancies.^[13,14]^ A PET/CT scan images the patient from the vertex of the skull to the toes including the upper extremities.^[14]^ These PET/CT imaging studies can be used opportunistically to measure patients’ BSA, to create new formulae for estimating BSA, and to evaluate the accuracy of the previously used formulae for BSA estimation. Interindividual variability in response to drug therapy is a well-known problem that may result in overtreatment and potential drug toxicity in some patients, and undertreatment and potential lack of therapeutic effect in other patients.^[15–17]^

We used machine learning with ridge regression for model building rather than ordinary least squares regression for several reasons. Ridge regression results in better prediction accuracy in unseen/new/real datasets and a lower mean squared error (MSE).^[18,19]^ This means that the model is likely to be more accurate than models built using ordinary least squares regression and will likely generalize to other datasets.^[18,19]^ Ridge regression handles multicollinearity extremely well,^[18,19]^ which is an expected problem because of the correlation between anthropometric measurements like height and weight. Ridge regression is known to prevent overfitting and result in more stable and reliable coefficients.^[18,19]^ Ridge regression is quite helpful when p > n (more predictors than observations), and useful in imbalanced datasets. Finally, ridge regression is rotationally invariant, so that the results will not be affected by how the predictors are scaled.^[18,19]^

The aims of this study are: to evaluate how age, height, weight, and BMI are associated with BSA; to create a new formula to estimate BSA using 3D PET/CT data; to compare the accuracy of this new formula to previously used formulae to estimate BSA.

## 2. Materials and methods

The study was approved by the Institutional Review Board at the Mayo Clinic, and the need for signed informed consent from each patient was waived. The data collection and analysis were compliant with the Health Insurance Portability and Accountability Act. This biomedical research followed the Declaration of Helsinki Ethical Principles for Medical Research principles for ethical biomedical research.

### 2.1. Patients

The study was comprised of adult patients (18 years or older) who had surveillance whole-body (vertex to toes including the entire upper extremities) PET/CT scans performed at the Mayo Clinic between 01/01/2010 and 12/31/2023. Patients were included if they had: the entire body and extremities included in the field of view; no active malignancy for over 1 year; and no evidence of active disease on PET/CT. Patients were excluded if: the field of-view truncated some part of the body; or if they had active malignancy/disease recurrence or were currently being treated for an active malignancy. We excluded patients with active malignancy because cancer cachexia affects both muscle and fat^[20,21]^ and could result in BSA estimates that are not generalizable to the general population.

Patient age, sex, self-reported race and ethnicity, height, weight, and BMI, at the time of the whole-body PET/CT scan were recorded.

### 2.2. PET/CT

PET/CTs were performed using a General Electric (GE Healthcare, Waukesha, WI) PET/CT scanner. CT scans were obtained for attenuation correction at 120 kVp, variable mA, and slice thickness of 5 mm. Patients were required to fast for 8 hours prior to the procedure. Fasting blood glucose levels had to be below 200 mg/dL. Patients were injected with 8–20 mCi (296–740 MBq) of ^18^F-fluorodeoxyglucose (^18^F-FDG) into a vein in the antecubital fossa. Patients were then instructed to rest for approximately 60 minutes in a quiet dark room, and then the PET images were obtained.

### 2.3. CT

CT scans were performed using a Siemens (Siemens Healthineers, Erlangen, Germany) Somatom Definition Edge and Siemens Definition Flash scanners, 120–140 kVp, variable mAs, and tilt 0^0^. Approximately 150 ml of Iohexol 350 mg/ml (Ominipaque 350, GE Healthcare, Waukesha, WI) was administered intravenously for all CT scans. CT images were obtained approximately 75 seconds after intravenous contrast administration.

### 2.4. Data processing

Whole-body ^18^F-FDG PET/CT scans were downloaded from the Picture Archiving and Communications System in digital imaging and communications in medicine format. Non-deformational registration was used to fuse the CT component of the ^18^F-FDG PET/CT scans when the scans were completed as 2 separate series.

### 2.5. Segmentation

**Figure 1.**
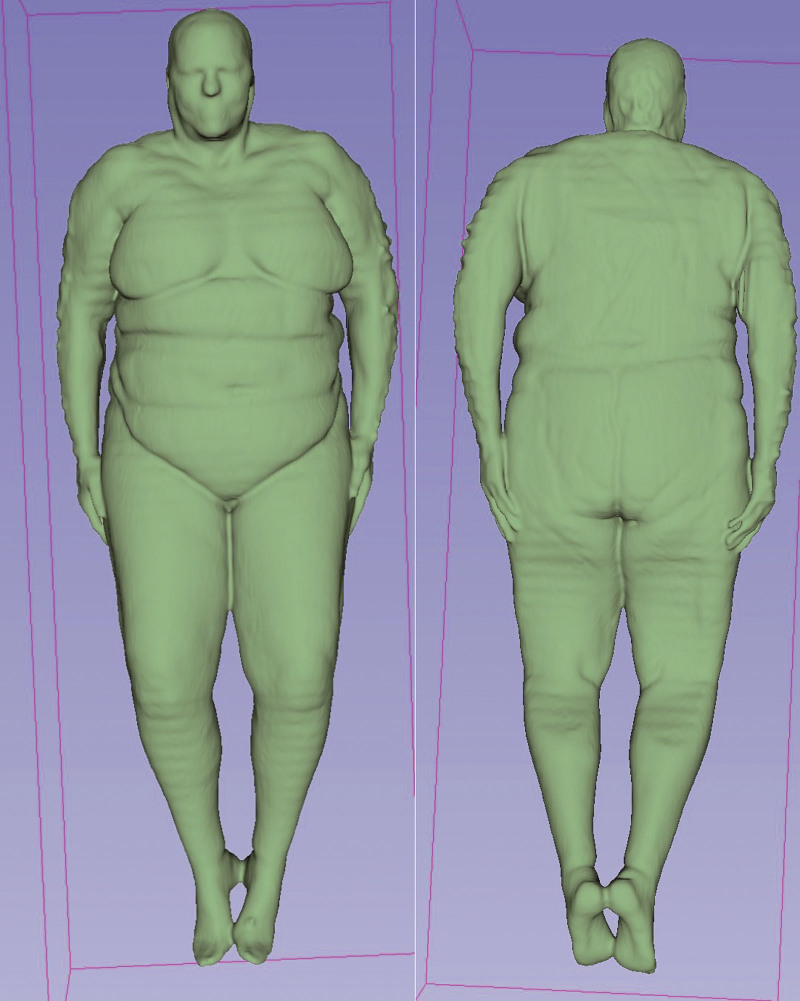
Segmentation of the total body surface area from whole-body CT. CT = computed tomography.

Digital imaging and communications in medicine sequences were uploaded into 3D-Slicer.^[22]^ We used the freely available TotalSegmentator toolkit^[23]^ to complete segmentations. TotalSegmentator was used to automatically segment the total BSA from the CT component of the PET/CT (Fig. 1).^[23,24]^ All segmentations were performed by a trained imaging physician researcher and were reviewed and refined by a board-certified diagnostic radiologist with more than 10 years of experience.

### 2.6. Hardware

Segmentations were performed using a NVIDIA Quadro RTX 4000 (DRAM: 8 GB), central processing unit: Intel Xeon W-2123, 3.60 GHz, and random-access memory: 64 GB).

### 2.7. Statistics

#### 2.7.1. A priori sample size/power calculation

A sample size of 350 patients has 80% power to detect a BSA difference of 600 cm^2^ (0.06 m^2^) assuming the standard deviation of the BSA is 2000 cm^2^ (0.2 m^2^).

#### 2.7.2. To evaluate how age, height, weight, and BMI are associated with BSA

Pearson correlation coefficients were used to evaluate how age, height, weight, and BMI are associated with BSA. This analysis was repeated stratified by race and sex. Fisher *z* test was used to compare correlations. The Bonferroni correction for multiple corrections were utilized, so that *P* values < .05/30 = .0017, were considered statistically significant.

#### 2.7.3. Creating a new formula to estimate BSA

The BSA was converted to m^2^, and then log-transformed, since BSA has been shown to have a log-normal distribution.^[10]^ We then used multivariate ridge regression with 5-fold cross-validation to predict BSA using age, height, weight, sex, and race as predictors. Ridge regression was chosen because it reduces model overfitting by adding an L2 penalty to the regression coefficients, shrinking them toward zero, which improves predictive accuracy and stability, especially in the presence of multicollinearity or high-dimensional data. It also maintains all predictors in the model, unlike subset selection methods, providing a more robust solution when many variables are relevant.

Age was removed from the model for several reasons. First, age was not a significant predictor of BSA in our dataset (*P* > .05). We analyzed adults, and BSA is not generally associated with age in adults. A large person could be 18 years of age, and a small person could be 90 years of age or vice versa. A person may gain or lose weight and change BSA at any given age. Finally, age was removed from the model because age was not used as a predictor in any of the previously published models.

Race was included in the model for 2 reasons. It is well-known that Blacks/African Americans have longer upper and lower extremities (limbs) than their White counterparts.^[25]^ In addition, they also have shorter torsos than their White counterparts.^[26]^ These changes are thought be evolutionarily derived and we are the first to investigate how these changes contribute to differences in BSA between races. Full model details are shown in Appendix 1, Supplemental Digital Content, https://links.lww.com/MD/R752.

#### 2.7.4. To compare the accuracy of our equation to previously used equations to estimate BSA

We evaluated 11 of formulas most used to estimate BSA.^[3–11]^ The mean absolute error (MAE) is a metric that quantifies the average magnitude of errors in a set of predictions, without considering whether the predictions are over or under the actual values. It is calculated by averaging the absolute differences between predicted BSA and actual measured BSA. MAE is commonly used in machine learning to assess the accuracy of a model’s predictions. For the BSA equations that did not adjust for sex, we evaluated whether the MAE was different for males and females. We compared the MAE between men and women adjusting for race, height, and weight, to see if the MAE was different for males and females. We also compared the MAE between races adjusting for sex, height, and weight to evaluate whether the MAE was different between races using *t* tests.

The MSE is a metric often used to evaluate the accuracy of a statistical model. It quantifies the average squared difference between the predicted values from the BSA formulas and the actual measured BSA values. A smaller MSE indicates a better-fitting formula, with predicted BSA values closely matching the actual measured BSA values. We repeated the analyses substituting the MSE for the MAE.

Statistics were calculated using Rv4.13 (https://www.r-project.org). All test statistics were 2-sided. P values less than the a priori Type I error rate of 0.05 were considered statistically significant.

## 3. Results

The study was comprised of a total of 698 adult patients (49.0% female) with ages ranging from 18 to 93 years. Approximately 3.7% (N = 26) were Black/ African American and 96.3% (N = 672) were White Americans. We found no significant differences in height, weight, and BMI between races, however, Blacks/African Americans were on average 10.6 years younger than White Americans in the study (Table 1).

**Table 1:** Demographic and anthropometric measurements.

Variable	All (N = 698)
Age, years	62.5 (15.8)
Height, m	1.70 (0.09)
Weight, kg	83.0 (21.4)
BMI, kg/m^2^	28.6 (6.4)
BSA, m^2^	1.85 (0.28)
Male	All (N = 356)
Age, years	65.5 (14.9)
Height, m	1.76 (0.07)
Weight, kg	90.2 (18.4)
BMI, kg/m^2^	28.9 (5.3)
BSA, m^2^	1.94 (0.24)
Female	All (N = 342)
Age, years	59.5 (16.2)
Height, m	1.64 (0.07)
Weight, kg	75.6 (21.8)
BMI, kg/m^2^	28.1 (7.4)
BSA, m^2^	1.76 (0.28)

BMI = Body Mass Index, BSA = body surface area, kg = kilograms, m = meters, N = number of individuals.

() = standard deviation.

### 3.1. To evaluate how age, height, weight, and BMI are associated with BSA

Table 2 shows the correlations between the anthropomorphic measurements and BSA. Height (*R* = 0.49, *P* < .001), Weight (*R* = 0.83, *P* < .001), and BMI (*R* = 0.72, *P* < .001) were positively correlated with BSA for all. Age was not correlated with BSA (*R* = 0.05, *P* > .05).

**Table 2:** Correlations between demographic and anthropometric measurements.

First variable	Second variable	All
Age	Height	−0.04
Age	Weight	0.01
Age	BMI	0.04
Age	BSA	0.05
Height	Weight	0.51
Height	BMI	0.09
Height	BSA	0.49
Weight	BMI	0.90
Weight	BSA	0.83
BMI	BSA	0.72
Male		
Age	Height	−0.22
Age	Weight	−0.09
Age	BMI	−0.01
Age	BSA	−0.01
Height	Weight	0.43
Height	BMI	0.07
Height	BSA	0.42
Weight	BMI	0.93
Weight	BSA	0.75
BMI	BSA	0.65
Female		
Age	Height	−0.25
Age	Weight	−0.03
Age	BMI	0.06
Age	BSA	−0.01
Height	Weight	0.38
Height	BMI	0.07
Height	BSA	0.36
Weight	BMI	0.94
Weight	BSA	0.85
BMI	BSA	0.80

BMI = body mass index, BSA = body surface area.

* - significant at the *P* < .05/30 = 0.0017.

In men, the analysis revealed positive correlations between BSA and height (*R* = 0.42, *P* < .001), weight (*R* = 0.75, *P* < .001), and BMI (*R* = 0.65, *P* < .001), but no correlation with age (*R* = -0.01, *P* > .05).

In women, height (*R* = 0.36, *P* < .001) and weight (*R* = 0.85, *P* < .001) were positively correlated with BSA, while age showed no correlation with BSA (*R* = -0.01, *P* > .05) (Table 2).

### 3.2. Creating a new formula to estimate BSA

We found the optimal formula to estimate BSA given height, weight, sex, and race is as follows:


$$\begin{array}{l} \begin{array}{lll} & BSA\;in\;m^{2}=\;0.2626943\;{\left(Height\;in\;m\right)}^{0.280306}\; & {\left(Weight\;in\;kg\right)}^{0.415072}\left(1.001269\;if\;Male\right)\left(0.96941\;if\;White\right)\; \end{array} \end{array}$$ [Equation 1]

### 3.3. To compare the accuracy of our formula to previously used formulae to estimate BSA

Several formulae most used to estimate BSA were evaluated (Table 3). We compared the MAE and MAE of our formula to other formulae to estimate BSA.

**Table 3:** Comparison of the mean absolute errors (MAE) and mean squared errors (MSE) for BSA formulas.

Equation	BSA Formula	All MAE (N = 698)	All MSE (N = 698)
Du Bois formula (n = 9)	$0.007184xW^{0.425}H^{0.725}$	0.15 (0.11)	0.034 (0.05)
Mosteller formula	$0.016667xW^{0.5}H^{0.5}$	0.16 (0.12)	0.041 (0.06)
Haycock formula (n = 81)	$0.024265xW^{0.5378}H^{0.3964}$	0.17 (0.13)	0.048 (0.07)
Gehan and George formula (n = 401; 130 older than 20 years)	$0.0235xW^{0.51456}H^{0.42246}$	0.17 (0.13)	0.046 (0.07)
Boyd formula (n = 197)	$0.03330xW^{\left(0.6157-0.0188xlog_{10}\left(W\right)\right)}H^{0.3}$	0.18 (0.14)	0.050 (0.07)
Fujimoto formula (n = 201)	$0.008883xW^{0.444}H^{0.663}$	0.14 (0.09)	0.027 (0.05)
Takahira formula (unknown)	$0.007241xW^{0.425}H^{0.725}$	0.16 (0.11)	0.037 (0.06)
Shuter and Aslani (n = 42)	$0.00949xW^{0.441}H^{0.655}$	0.14 (0.10)	0.030 (0.05)
Lipscombe formula (n = NA, no gold standard)	$0.00878108xW^{0.434972}H^{0.67844}$	0.15 (0.11)	0.035 (0.05)
Schlich formula for women (unknown)	$0.000975482xW^{0.46}H^{1.08}$	0.13 (0.10)	0.035 (0.06)
Schlich formula for men (unknown)	$0.000579479xW^{0.38}H^{1.24}$	0.14 (0.10)	0.033 (0.06)
Current analysis (342 women)	$0.2626943\;xH^{0.280306}W^{0.415072}$ (0.96941 if White)	0.12 (0.08)	0.022 (0.03)
Current analysis (356 men)	$0.2626943xH^{0.280306}W^{0.415072}$ (1.001269)(0.96941 if White)	0.12 (0.10)	0.025 (0.05)

BSA = body surface area, H = height in cm, MAE = mean absolute errors, MSE = mean squared errors, n = number of individuals used to derive the original equations, NA = Not applicable, W = weight in kg.

() = standard deviations.

The MAE for men was 0.12, 95% confidence interval (CI) (0.11, 0.13), and for women was 0.12, 95% CI (0.11, 0.13). The MSE for men was 0.025, 95% CI (0.02, 0.03), and for women was 0.022, 95% CI (0.019, 0.025). Our formula had the lowest MAE of all investigated formulae, with a MAE of 0.12, and was significantly better than the Dubois (*P* < .001), Haycock (*P* < .001), Gehan and George (*P* < .001), Boyd (*P* < .001), Fujimoto (*P* < .001), Takahira (*P* < .001), Shuter and Aslani (*P* < .001), Lipscombe (*P* < .001), and both Schlich formulae (*P* < .001) (Table 3). Our formula also had the lowest MSE of all investigated formulae and was significantly more accurate than all other formulae (*P* < .001) (Table 3).

No notable difference was observed in the unadjusted MAE between Black/African American and White American groups (*P* > .05 for all) (Table 3). Similarly, there was no significant difference in the unadjusted MSE between Black/African Americans and White Americans across all BSA estimation formulae (Table 3).

The final BSA formula shows that for the same height and weight, the BSA was 3.1% higher for Blacks/African Americans than White Americans (*P* = .032), and 0.13% higher for males than females (*P* < .05). We observed that, for equivalent height and weight, males exhibit slightly higher BSA than females, possibly due to variations in body contours associated with secondary sexual characteristics. After adjusting for sex, height, and weight, Black/African Americans showed significantly higher BSA compared to White Americans (*P* = .032).

## 4. Discussion

This analysis showed that weight was positively correlated with, and the strongest predictor of BSA. Height was also positively correlated with BSA. There was no association between BSA and age. We used multivariate ridge regression with 5-fold cross-validation to create a robust formula to estimate BSA using 698 individuals. On average, our formula results in < 1.5% MSE for BSA estimation. This formula was more accurate (resulted in lower MAE and lower MSE) than the Dubois, Haycock, Gehan and George, Boyd, Fujimoto, Takahira, Shuter and Aslani, Lipscombe, and both Schlich models.^[3–11]^ Men had slightly higher BSA than women after adjusting for height and weight. We found that Blacks/ African Americans had 3% higher BSA than White Americans after adjusting for sex, height, and weight.

BSA has been shown to be positively correlated with height, weight, and BMI,^[5–11]^ which supports our findings. Schlich created separate formulae for males and females suggesting that better BSA estimates were possible if the formulae were restricted by sex.^[11]^ We also found that creating separate formulae for each sex resulted in better BSA estimates. Other formulae exist for estimation of BSA. The Dubois formula is arguably the most used formula but was based on measurements made on only 9 individuals of unknown sex, racial and ethnic diversity.^[3]^ The Haycock formula was derived based on 81 subjects, and estimates BSA by modeling the body as a cylinder and the head as a sphere.^[5]^ Gehan and George evaluated 401 subjects.^[6]^ Of these 401 subjects, the BSA was estimated by surface coating for 163 subjects; by surface integrator for 222 subjects; and using triangulation for 16 subjects.^[6]^ Only 130 of the 401 subjects were adults aged 20 years or older, so the adult population in this study is substantially smaller than ours.^[6]^ Boyd created a BSA formula based on analysis of 197 subjects, but it is unclear what was the age, sex, race and ethnicity of these subjects.^[7]^ Fujimoto BSA formula was based on 201 adult and children Japanese subjects, which limits the generalizability of their BSA formula for non-Japanese subjects.^[8]^ Shuter and Aslam created a formula for estimating BSA based on 42 subjects using simple multivariable linear regression with no cross-validation.^[9]^ The sex, race, and ethnicity of these subjects are unknown.^[9]^ Our method used cross-validation which is a technique that is known to improve the overall performance of the formula. Lipscombe created a formula based on the geometric mean of 22 preexisting formulas for BSA estimation.^[10]^ Lipscombe formula works reasonably well but had higher MAE and MSE than our own formula. Schlich analyzed data from 188 subjects, of which 7 were children, 132 were women and 49 were men to estimate BSA.^[11]^ The race and ethnicity of these subjects were not recorded, so it is unclear how well this formula performs in diverse populations. The Schlich formulae also have higher MSE and MAE than our formulas. We noted that the addition of sex and race as variables when coupled with machine learning techniques (ridge regression) resulted in more accurate prediction of BSA.

Males had slightly higher BSA than women for a given height and weight. Men may have higher BSA than women for a given height and weight due to differences in body composition and morphology. Men tend to have a higher proportion of lean muscle mass and longer limbs relative to their torso, which increases their BSA, whereas women typically have a higher proportion of body fat and relatively shorter limbs, resulting in a slightly lower BSA. Blacks/African Americans had a slightly higher mean BSA than White Americans, after adjusting for sex, height, and weight, likely due to differences in body morphology such as relatively shorter torsos and longer extremities. This variation may be influenced by evolutionary adaptations to climate, with tropical origins favoring higher BSA for heat loss in Blacks/African Americans, compared to lower BSA for heat conservation in White Americans from temperate climates.^[27–30]^

BSA is primarily used for 3 things in clinical medicine: for estimating drug doses, for patients with burns, and for dialysis. Some drugs, for example chemotherapy drugs, are dosed based on BSA. Several drugs have a narrow therapeutic index, including aminoglycosides, cyclosporin, carbamazepine, digoxin, digitoxin, flecainide, lithium, phenytoin, phenobarbital, and warfarin, which means that there is a small difference between their therapeutic and toxic doses.^[31,32]^ Therefore, these findings need to be further explored to understand their potential clinical significance. For patients with burns—the % BSA affected by the burn is used to determine prognosis, to determine the level of care necessary and whether to transfer to a burn center, to determine the patient’s calorific needs, and to determine rate of fluid resuscitation. For a patient on dialysis, BSA is used is used to determine how big the dialyzer should be, to determine the blood flow and dialysis fluid to use, and to assess whether the dialysis dose is sufficient for the patient. Our goal was to create more accurate and reliable models for estimating BSA. It is unclear how small changes in BSA would have a measurable impact on patient outcomes, so further research is needed in this area.

The study has a few limitations. The patients all come from a tertiary care academic center with main sites in 3 very different geographic states (Rochester, Minnesota; Scottsdale, Arizona; and Jacksonville, Florida). The patients had a history of cancer but have been disease free for at least 1 year, so it is unlikely that prior treatment changes could affect current BSA. The dataset is imbalanced and lacks racial diversity with 26 Black/African American (3.7%) and 672 White (96.3%) participants. To counteract this, we used ridge regression, which is best for imbalanced datasets and results in stable and reliable coefficients and produces models with better prediction accuracy in this setting.^[18,19]^ We did not have an independent external validation dataset; however, we note that none of the previously published models used to estimate BSA used machine learning methods, cross-validation methods, or even as large a dataset as we used. To counteract this, we used ridge regression, which is one of the best techniques for ensuring prediction accuracy in unseen/new/real datasets.^[18,19]^ The race result is preliminary and needs validation in a larger, more balanced group. Finally, the formulae were developed in adult subjects so may not generalize to the pediatric population.

In summary, we created new formulae for BSA estimation and show that these formulae provide more accurate estimates with lower MSE and lower MAE than the currently available formulae.

**Key Points**

- Using multivariable ridge regression with cross-validation, we analyzed whole-body CT imaging data to develop new formulae for estimating body surface area (BSA) based on sex, height, and weight.
- These novel formulae outperform existing BSA estimation methods, demonstrating lower mean squared error (MSE) and mean absolute error (MAE).
- Our approach enhances the accuracy of BSA estimates, offering a more reliable tool for clinical and pharmacological applications.

## Author contributions

**Conceptualization:** Ronnie Sebro.

**Data curation:** Mahmoud Elmahdy.

**Formal analysis:** Ronnie Sebro, Mahmoud Elmahdy.

**Methodology:** Ronnie Sebro.

**Resources:** Ronnie Sebro.

**Supervision:** Ronnie Sebro.

**Writing – original draft:** Ronnie Sebro, Mahmoud Elmahdy.

**Writing – review & editing:** Ronnie Sebro, Mahmoud Elmahdy.

## Supplementary Material


