# Supplementary material for: Artificial intelligence-driven body surface area (BSA) estimation using computed tomography: Comparative evaluation with existing formulae
Source: Medicine (Baltimore). 2026 Apr 24;105(17):e48478. doi: 10.1097/MD.0000000000048478 (PMC13124374; doi:10.1097/MD.0000000000048478)
Supplement: Supplementary file 1 [file medi-105-e48478-s001.pdf]

Supplementary Material
Appendix I

We used the glmnet (glmnet()) package in R to perform multivariable ridge regression. This package is designed for fitting regularized regression models, including ridge regression. By default, glmnet standardizes the predictors internally. This ensures fair penalization in ridge regression. We set alpha = 0 for ridge regression (L2 penalty). The lambda parameter controls the strength of regularization. We used 5-fold cross-validation to select the best lambda value using the cv.glmnet() function. We plotted the cross-validation curve to inspect the performance across different lambda values. Finally, the fitted model with the optimal lambda was used to extract coefficients.
